# Effectiveness of PEER Intervention on Older Adults’ Physical Activity Time Series Using Smoothing Spline ANOVA

**DOI:** 10.3390/math13030516

**Published:** 2025-02-04

**Authors:** Yi Liu, Chang Liu, Liqiang Ni, Wei Zhang, Chen Chen, Janet Lopez, Hao Zheng, Ladda Thiamwong, Rui Xie

**Affiliations:** 1 Department of Statistics and Data Science, University of Central Florida, Orlando, FL 32816, USA;; 2 Department of Computer Science, University of Central Florida, Orlando, FL 32816, USA;; 3 College of Nursing, University of Central Florida, Orlando, FL 32816, USA;; 4 Department of Electrical and Computer Engineering, University of Central Florida, Orlando, FL 32816, USA;; 5 Disability, Aging and Technology Cluster, University of Central Florida, Orlando, FL 32816, USA

**Keywords:** smoothing spline ANOVA, physical activity, accelerometer, wearable devices, time series

## Abstract

Falls are a major cause of injury among older adults. The Physio-fEedback Exercise pRogram (PEER) combines physio-feedback, cognitive reframing, and guided exercises to reduce fall risk. However, its impact on physical activity (PA) over time is underexplored. Functional time-series analysis offers insight into behavior patterns and sustainability. This preliminary study assessed PEER’s effectiveness in improving PA levels immediately and over time. A total of 64 community-dwelling older adults were cluster-randomized into PEER (N=33) or control groups (N=31). Participants wore Fitbit trackers, generating time-series data on activity. The PEER group completed an 8-week program, while the control group received CDC fall prevention pamphlets. PA data were analyzed using smoothing spline analysis of variance (SSANOVA), chosen for its flexibility in modeling complex, non-linear relationships in time-series data and its ability to handle skewed distributions and repeated measures. Unlike traditional parametric models, SSANOVA decomposes temporal trends into interpretable components, capturing both smooth trends and abrupt changes, such as those occurring on group workout days. This capability ensures robust and nuanced analysis of intervention effects. Results showed PEER participants significantly increased evenly and had very active minutes and reduced sedentary behavior during the intervention. No significant effect was found for light active minutes. Specifically, during the intervention period, PEER participants engaged in an average of 6.7% fewer sedentary minutes per day, 13.8% additional fairly active minutes per day, and 2.8% additional very active minutes per day compared to the control group. While the reduction in sedentary minutes and increase in fairly active minutes were not statistically significant, the increase in very active minutes was significant. However, our functional time-series analysis revealed these improvements diminished over the 15-week follow-up, indicating challenges in maintaining PA. In conclusion, PEER boosts PA and reduces sedentary behavior short-term, but strategies are needed to sustain these benefits. In conclusion, PEER boosts PA and reduces sedentary behavior short-term, but strategies are needed to sustain these benefits. Public health policies should emphasize technology-driven fall risk assessments, community-based prevention programs, and initiatives that promote physical activity, home safety, and chronic condition management.

## 1. Introduction

### 1.1. Background and Motivation

Falls are the leading cause of injury and injury-related deaths among adults aged 65 and older in the United States [1]. Statistics show that an older adult dies from a fall every 19 min [2], and approximately one-third of community-dwelling older adults experience a fall each year [3]. Falls often lead to a fear of falling, which reduces physical activity (PA) levels [4]. This decline in PA contributes to chronic conditions and increases the risk of future falls [5,6]. Community-dwelling older adults, especially those with low income, experience unique challenges, including environmental factors, lack of caregivers, and individual differences in health, that contribute to fall risk [7–9]. These populations also often face challenges such as limited access to technology, low digital literacy, and socioeconomic constraints that hinder their ability to engage with and benefit from technology-based interventions [10,11]. Addressing these risks and intervention gaps in community settings is critical to developing effective and tailored interventions that promote safety and independence while reducing the healthcare burdens associated with falls [12].

Exercise interventions have been shown to reduce fall rates [13]. The US Preventive Services Task Force recommends such interventions for fall prevention among community-dwelling older adults [1]. Nurse-led fall prevention programs have also reduced fall rates in hospitals [4]. However, there is a lack of sufficient research on the effectiveness of technology-enhanced, feedback-based exercise programs, especially for underserved, low-income populations [14–16]. Previous studies have used accelerometer-measured PA to explore fall risk categories. For example, higher levels of PA, as measured by accelerometers, were associated with a substantially reduced risk of falling in older adults [17,18]. In addition, participants with high daily active minutes had a lower risk of falling [19].

### 1.2. Literature Review

Recent advancements in mobile health (mHealth) technologies have shown great promise in promoting PA among older adults [20]. Wearable devices, such as accelerometers and fitness trackers, can monitor activity levels, provide real-time feedback, and encourage regular exercise [21]. Studies indicate that technology-enhanced interventions, including apps and wearable devices, improve adherence to exercise regimens by offering continuous feedback and reinforcing behavior change [3,15,16]. These interventions are particularly beneficial for older adults who face barriers to attending in-person sessions or who live in remote areas [22]. Nonetheless, significant gaps remain in understanding how these interventions can effectively increase PA, particularly among low-income and high-risk populations [14,15]. The Physio-fEedback Exercise pRogram (PEER) intervention is unique compared to other programs due to its integrative approach, which combines physio-feedback, cognitive reframing, and guided exercises [5,6]. This multifaceted design addresses physical and psychological factors that contribute to fall risks, including fear of falling and self-efficacy enhancement. Previous research has explored fall risk factors and their interrelationships across multiple domains, including sociodemographic characteristics, mental well-being, body composition, self-assessed and performance-based fall risk assessments, and PA patterns [23]. However, there is limited research evaluating the effectiveness of technology-integrated interventions, such as PEER.

In addition, while common statistical methods, such as linear regression and descriptive-based analyses, are frequently applied to accelerometer data [24,25], they often fall short of capturing the complex, non-linear temporal patterns and inherent variability of physical activity data. Analyzing time-series data from accelerometer-based PA monitoring presents unique challenges, including non-linear relationships, high variability, and heterogeneity in large datasets [26]. Generalized Additive Models (GAMs) offer flexibility in modeling nonlinear relationships by additively summing smooth functions of predictors [27]. Linear or generalized mixed-effects models are adept at handling grouped or longitudinal data by incorporating random effects to account for within-group or temporal correlations [28], yet they often rely on parametric assumptions that may not fully capture the intricate temporal dynamics present in physical activity data. While frameworks like the individualized dynamic model [29] can effectively handle longitudinal data with low resolution, they fall short in addressing the complexities of accelerometer data. This study aims to tackle these challenges by leveraging advanced statistical approaches tailored to the data’s time-series nature, providing a robust evaluation of the PEER intervention’s effectiveness in promoting physical activity levels among older adults.

### 1.3. Study Framework

This preliminary study analyzes time-series data collected during an ongoing parallel, two-arm clustered randomized controlled trial, “Optimizing a technology-based body and mind intervention to prevent falls and reduce health disparities in low-income populations”, with the primary aim of examining differences in fall risk and PA following completion of the PEER compared to the control group among community-dwelling older adults [19].

In this work, we propose using the smoothing spline analysis of variance framework [30,31] to analyze the temporal patterns and inter-group differences of the PA data. Smoothing spline is an asymptotic and empirical approach that is especially advantageous when applied to large-scale data, as demonstrated in various previous works [32–34]. Studies using SSANOVA have demonstrated its strength in handling cyclic biomechanical data [35] and accelerometer-based PA data across different fall risk groups [36]. Ma et al. [37] extend SSANOVA’s application through adaptive basis selection for exponential family smoothing splines, which is particularly useful for joint modeling of multiple sequencing samples.

SSANOVA is particularly well-suited for time series data, as it decomposes temporal trends into interpretable components while handling variability across repeated measures and skewed distributions [38]. In this study, SSANOVA is used to uncover both immediate and longer-term intervention effects by modeling daily activity patterns over time, offering a robust framework for comparing PEER and control groups [31,36]. In addition, unlike traditional parametric models, SSANOVA can capture both smooth trends and abrupt changes [39], such as those occurring during group workout days, making it an ideal framework for studying the dynamic effects of the PEER intervention.

### 1.4. Contributions

In this paper, we show the benefits of using SSANOVA to model the effectiveness of the PEER intervention over time, leveraging the time-series nature of the data. The impact of PEER on physical activity has been underexplored, as it integrates multiple components to reduce fall risk. We apply SSANOVA to assess the effectiveness of PEER, capturing PA trends over time [19,31]. This method provides smooth, flexible representations of evolving PA patterns without assuming rigid distributions and accommodates different distributions across categories (e.g., sedentary, light, fairly, and very active minutes) [30,36]. Additionally, SSANOVA captures non-linear intervention effects over time [32,33,40]. Our analysis highlights several key advantages:

- We evaluate the effectiveness of PEER, introduced by Thiamwong et al. [19], an integrative intervention that blends physio-feedback, cognitive reframing, and guided exercises to reduce sedentary behavior and increase moderate-to-vigorous physical activity in older adults.
- We use an SSANOVA approach to model non-linear trends and distributional differences in PA data over time. This flexible and robust method helps us navigate the complexity of minute-level PA data without imposing parametric assumptions.
- Our work also shows that SSANOVA is particularly effective in evaluating intervention programs like PEER by highlighting the impact of key intervention days and revealing how specific activities shift PA patterns over time.

## 2. Methods

### 2.1. Participants

PEER is an individualized intervention that includes three steps [19]: (1) technology-based physio-feedback using a real-time portable technology BTracks Balance System (BBS), (2) cognitive reframing based on Fall Risk Appraisal (FRA) matrix [6], and (3) PEER-led exercise that focuses on strength and balance training. The study utilized a two-arm clustered randomized trial design, as we are using community-dwelling older adults clustered into naturally occurring units: the communities. The two-arm design refers to the PEER intervention group versus the control group. Such a design allows us to study the effectiveness of interventions in real-world group settings while reducing the risk of contamination between the experimental and control groups.

**Figure 1.**
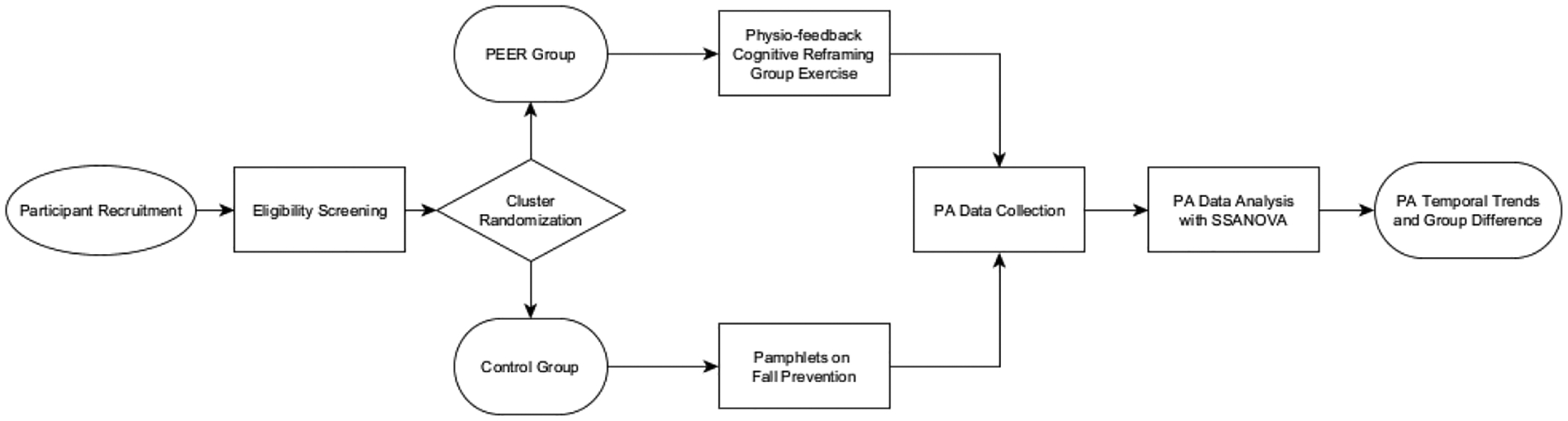
Flowchart of the study design and methodology.

Participants were recruited from 11 independent living communities and senior centers in Orlando, FL (This study utilizes data from two of the 11 sites that have completed the study), through flier distribution, word-of-mouth, and community partners facilitating our introduction, with the following inclusion/exclusion criteria: the inclusion criteria comprise an (1) age of 60 years or older, (2) being able to walk with or without an assistive device (but without the assistance of another person), (3) no marked cognitive impairment (Memory Impairment Screen score ≥ 5); (4) fluency in English or Spanish, and (5) living in their own homes or apartments. The exclusion criteria are as follows: for the PEER group, (1) a medical condition precluding exercise such as uncontrolled cardiac disease (shortness of breath or feeling pressure, squeezing, burning, or tightness when performing a PA), or (2) currently receiving treatment from a rehabilitation facility. For the control group: currently receiving treatment from a rehabilitation facility. Participants that meet the above criteria are assigned to either the PEER or control group through cluster randomization design, with senior living sites serving as clusters. The study spans six months and data were collected at four time points: baseline (T1), program completion (T2), and follow-ups at 3 months (T3) and 6 months (T4). Figure 1 provides a visual summary of the study design and methodology, including participant recruitment, randomization, intervention components, data collection, and data analysis. This paper focuses on accelerometer-based PA data from baseline to the end of T3, spanning 23 weeks, to assess how PA changes over time. PA data from the first two completed sites are used in this study. The total sample size is 64 participants, with 33 in the PEER group and 31 in the control group. A summary of the participant characteristics is shown in Table 1.

### 2.2. PEER Intervention

The Physio-fEedback Exercise pRogram (PEER) is an intervention program led by a trained PEER coach that focuses on balance, strength training, and exercise adherence (one participant out of each PEER intervention group was selected and trained as the PEER coach to lead group exercise classes) [19]. It combines the concepts of physio-feedback, cognitive reframing, and exercise by motivating a shift in self-estimation of fall risk to align with physiological fall risk to improve balance and muscle strength and prevent falls. Group exercise training comprises four sets of exercises: (1) seated warm-up; (2) seated and standing strength training of upper and lower body; (3) standing and moving balance training; and (4) stretch of upper and lower body.

PEER participants take part in an eight-week, PEER-led group exercise once a week as described above, and perform the same sets of individual exercises at home for at least 30 min per session, twice weekly. In contrast, participants in the control group receive information brochures on falls, simple exercises for balance improvement, and fall prevention in older adults developed by the Central Disease Control (CDC). Daily PA is tracked by Fitbit fitness trackers worn on the non-dominant hand by all participants throughout the study duration.

### 2.3. Physical Activity Data

Each participant was given a Fitbit Charge 5 (FB421) [41] device to wear on their dominant wrist for continuous activity tracking, generating time-series data throughout the study period. Real-time Fitbit data were streamed to the Fitbit server, and team members used APIs from the Fitbit developer account to download the data programmatically at regular intervals. The PA data were structured as a time series, capturing daily fluctuations in four categories: sedentary minutes, light active minutes, fairly active minutes, and very active minutes. Fitbit categorizes these activity levels based on metabolic equivalents (METs). Sedentary minutes: very low levels of movement, such as sitting or lying down (<1.5 METs). Light active minutes: light activities, including casual walking, cooking, or light household chores (1.5–3 METs). Fairly active minutes: moderate activities like brisk walking, gardening, or activities that raise the heart rate but are not strenuous (3–6 METs). Very active minutes: vigorous activities, such as running, cycling, or high-intensity aerobic exercises (>6 METs).

Participants were instructed to wear the Fitbit for the entire 6-month study period, allowing for continuous collection of time-series data. This paper focuses on data collected from baseline to prior time point 4 (T1–T3), spanning a 23-week period that includes both the 8-week intervention and the 13-week follow-up, the accelerometer generates one data point per day (the minutes spent in each activity levels for the day), resulting in a total number of 147 data points for each participant. The continuous time-series data allow for the analysis of short-term trends during the intervention and long-term behavior changes during the follow-up.

For potential Fitbit data accuracy and adherence issues, the SSANOVA model can handle missing values by automatically smoothing out adjacent time points, which ensures robust trend estimation [42,43]. The smoothing penalty in the model minimizes the impact of outliers and measurement errors in the data. Additionally, SSANOVA provides uncertainty quantification, enabling the estimation of trends with confidence intervals, which accounts for variability introduced by adherence inconsistencies. These features ensure that overall trends and intervention effects can still be reliably assessed, despite occasional gaps or inaccuracies in the raw Fitbit data.

### 2.4. Smoothing Spline Analysis of Variance

For the temporal pattern analysis of PA, we employed the smoothing spline ANOVA (SSANOVA) model due to its flexibility in modeling complex, non-linear relationships over time, regardless of whether the data follow a Gaussian distribution [30]. It flexibly decomposes temporal patterns into interpretable components while accounting for the intricate structure of repeated measures.

The SSANOVA model takes the general form of

(1) $$y_{i}=\eta \left(x_{i}\right)+\epsilon_{i},\;i=1,\cdots ,n,$$

where $y_{i}$ is recorded PA time series for i-th participant, and the covariates $x_{i}={\left(t_{i},g_{i}\right)}^{T}$, including time $t_{i}$ and treatment group $g_{i}$ (i.e., PEER vs. control). The random errors $\epsilon_{i}$ are assumed to be independent and identically distributed, η is the function to be estimated that represents the relationship between the covariates (time and group) and PA. By allowing for a flexible, data-driven function η, SSANOVA is well-suited to handle the inherent variability in accelerometer-based physical activity data.

SSANOVA decomposes the smoothing function $\eta \left(x_{i}\right)=\eta \left(t_{i},g_{i}\right)$ into components associated with time $t_{i}$ and group $g_{i}$ as,

(2) $$\eta \left(t_{i},g_{i}\right)=\eta_{0}+\eta_{1}\left(t_{i}\right)+\eta_{2}\left(g_{i}\right)+\eta_{12}\left(t_{i},g_{i}\right),$$

where $\eta_{0}$ is the constant function, $\eta_{1}$ represents the main effect of time, $\eta_{2}$ represents the main effect of group, and $\eta_{12}$ represents the interaction effect between time and group. This SSANOVA decomposition allows for estimating not only the overall temporal trends and group effects but also the interaction between time and group, which is critical for understanding the differential impact of the intervention.

**Figure 2.**
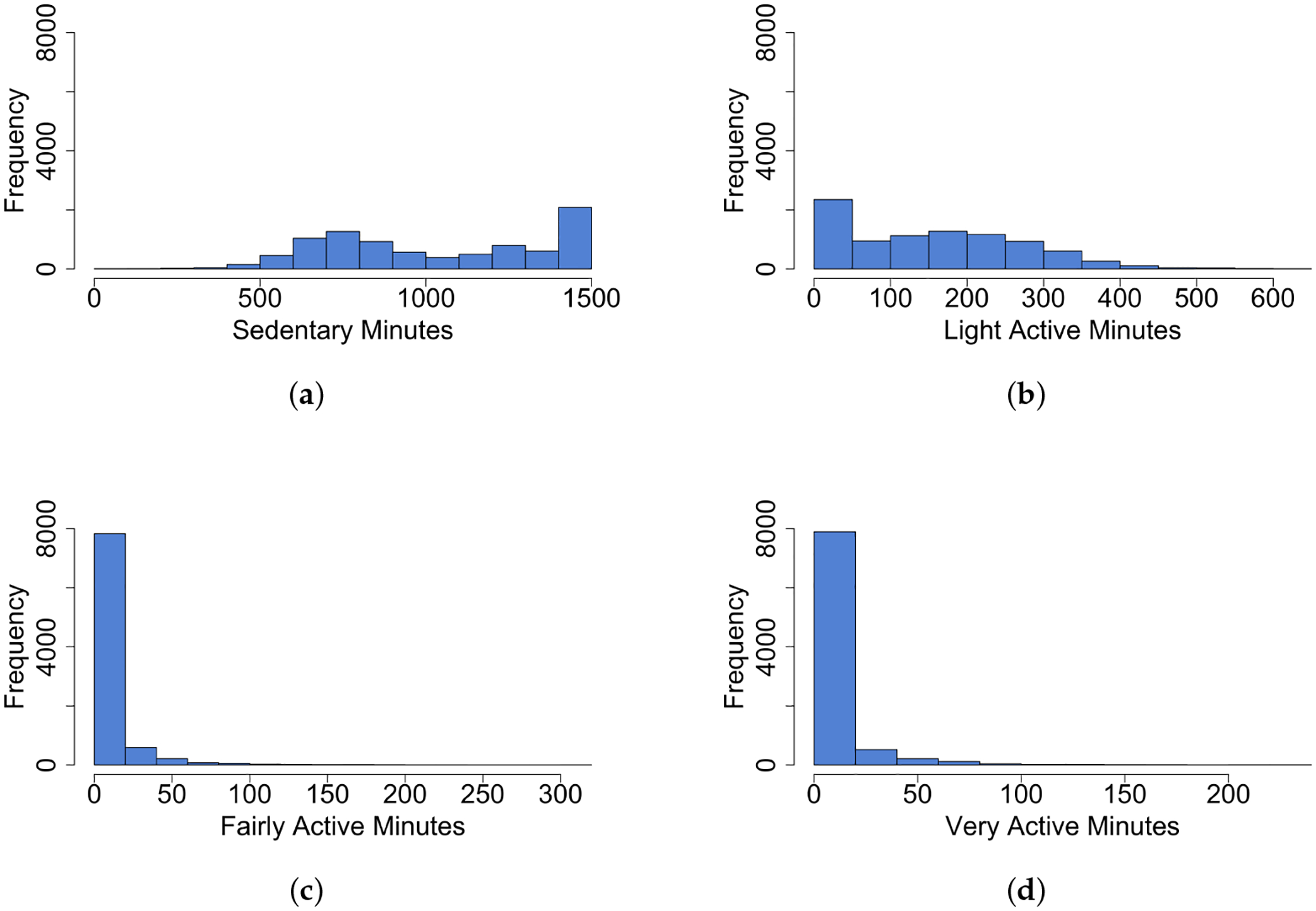
Marginal distribution (histogram) of 4 PA minutes categories. **(a)** Sedentary minutes; **(b)** light active minutes; **(c)** fairly active minutes; **(d)** very active minutes.

For sedentary and light active minutes, the Gaussian model was utilized, even though the data distributions are slightly skewed, as shown in Figure 2a,b. To ensure compatibility with the model, the response variables (sedentary minutes and light active minutes) were mean-adjusted to center the data around zero. The function η is estimated by minimizing the penalized least squares criterion,

(3) $$\frac{1}{n}\sum_{i=1}^{n}{\left(y_{i}-\eta \left(x_{i}\right)\right)}^{2}+\lambda J\left(\eta \right),$$

where J(⋅) is the roughness penalty and λ is the smoothing parameter that controls the trade-off between the goodness of fit and the smoothness of the estimated functions [30]. The optimal smoothing parameter is chosen through a generalized cross-validation strategy [44,45]. This approach ensures that the model adequately captures underlying patterns without overfitting the data. By selecting the optimal smoothing parameter, the SSANOVA model captures the temporal pattern and estimates group differences smoothly.

In contrast, for data types that exhibit significant skewness, including fairly active (Figure 2c) and very active minutes (Figure 2d), we use the Gamma model to accommodate the right-skewed nature of these activity levels. The Gamma distribution effectively models these non-normal distributions, ensuring that our analysis captures the true variability and trends within these more intense PA categories. We consider the Gamma distribution,

(4) $$f(y)=\frac{1}{\beta^{\alpha }\Gamma (\alpha )}y^{\alpha -1}e^{-y/\beta },$$

with α,β,y>0. Parallel to (3), we used the penalized likelihood functional

(5) $$-\frac{1}{n}\sum_{i=1}^{n}\left\{y_{i}e^{-\eta \left(x_{i}\right)}+\eta \left(x_{i}\right)\right\}/\sigma^{2}+\frac{\lambda }{2}J(\eta ),$$

with the log link η=log(αβ), and dispersion parameter $\alpha =1/\sigma^{2}$.

The SSANOVA model assumes independent and identically distributed (i.i.d.) random errors, a standard technical assumption in almost all statistical models [46,47]. The flexibility of SSANOVA enables the accommodation of non-i.i.d. data through appropriate modifications [30]. However, temporal correlations introduced by repeated measures within individuals, as well as clustering from grouping participants into the PEER intervention and control groups, can be incorporated into the SSANOVA framework by using correlation models, including random effects. Moreover, sedentary and light active minutes were mean-adjusted, while a Gamma distribution was applied to fairly and very active minutes, which exhibited significant skewness, accurately capturing variability and trends. These preprocessing steps, combined with a careful study design, effectively minimized potential bias and dependency, supporting the validity of the i.i.d. assumption and reinforcing the suitability of the SSANOVA framework for analyzing accelerometer-based PA data.

In both Gaussian and Gamma scenarios, the 95% Bayesian confidence intervals (CI) [30] were calculated in addition to the estimated function to assess the uncertainty of the model estimation. The flexibility of SSANOVA allows it to simultaneously account for linear and non-linear trends, interaction effects, and varying distributions in the data. This capability makes it particularly suitable for detecting significant temporal patterns and group differences in PA minutes, where traditional parametric models may fall short [44,45].

### 2.5. Semi-Parametric Modeling with Partial Splines

To capture the effect of specific days associated with the PEER-led group exercise, where we expected to see discrete jumps or shifts in the otherwise smooth function, we applied a partial spline model. This was preformed by adding a parametric covariate with an associated coefficient to the classical SSANOVA model [30]:

(6) $$y_{i}=z(t{)}_{i}^{T}\beta +\eta \left(x_{i}\right)+\epsilon_{i},\;i=1,\cdots ,n,$$

where z(t) is the parametric covariate indicating whether the day t corresponds to PEER-led group exercise day $\left(z(t{)}_{i}=1\right)$ or a non-group exercise day $\left(z(t{)}_{i}=0\right)$. Coefficient β quantifies the mean difference in PA levels between intervention and non-group intervention days. The magnitude of β reflects the extent of this difference, with a positive β indicating minutes spent in corresponding PA level are higher on group exercise days and negative β suggesting minutes spent in corresponding PA level are lower on group exercise days. And x is the nonparametric covariate. β and η are estimated through minimizing of

(7) $$\frac{1}{n}\sum_{i=1}^{n}{\left(y_{i}-z(t{)}_{i}^{T}\beta -\eta \left(x_{i}\right)\right)}^{2}+\lambda J(\eta ).$$

The resulting semi-parametric model allows for more flexibility by combining smooth splines for the temporal trends with fixed effects for the group exercise days.

Partial splines add interpretability and robustness to the original nonparametric model. The parametric term isolates the effect of group exercise days, enabling clear insights into the direct impact of the PEER program. It allows for the estimation of smooth trends while accounting for discontinuities and shifts associated with group exercise days.

## 3. Results

Our results showed that the PEER-led group exercise intervention demonstrated significant immediate benefits by increasing fairly active and very active minutes and reducing sedentary time during the 8-week intervention period compared to the control group. However, the effects appear to attenuate across all activity levels during the 13-week follow-up period.

**Figure 3.**
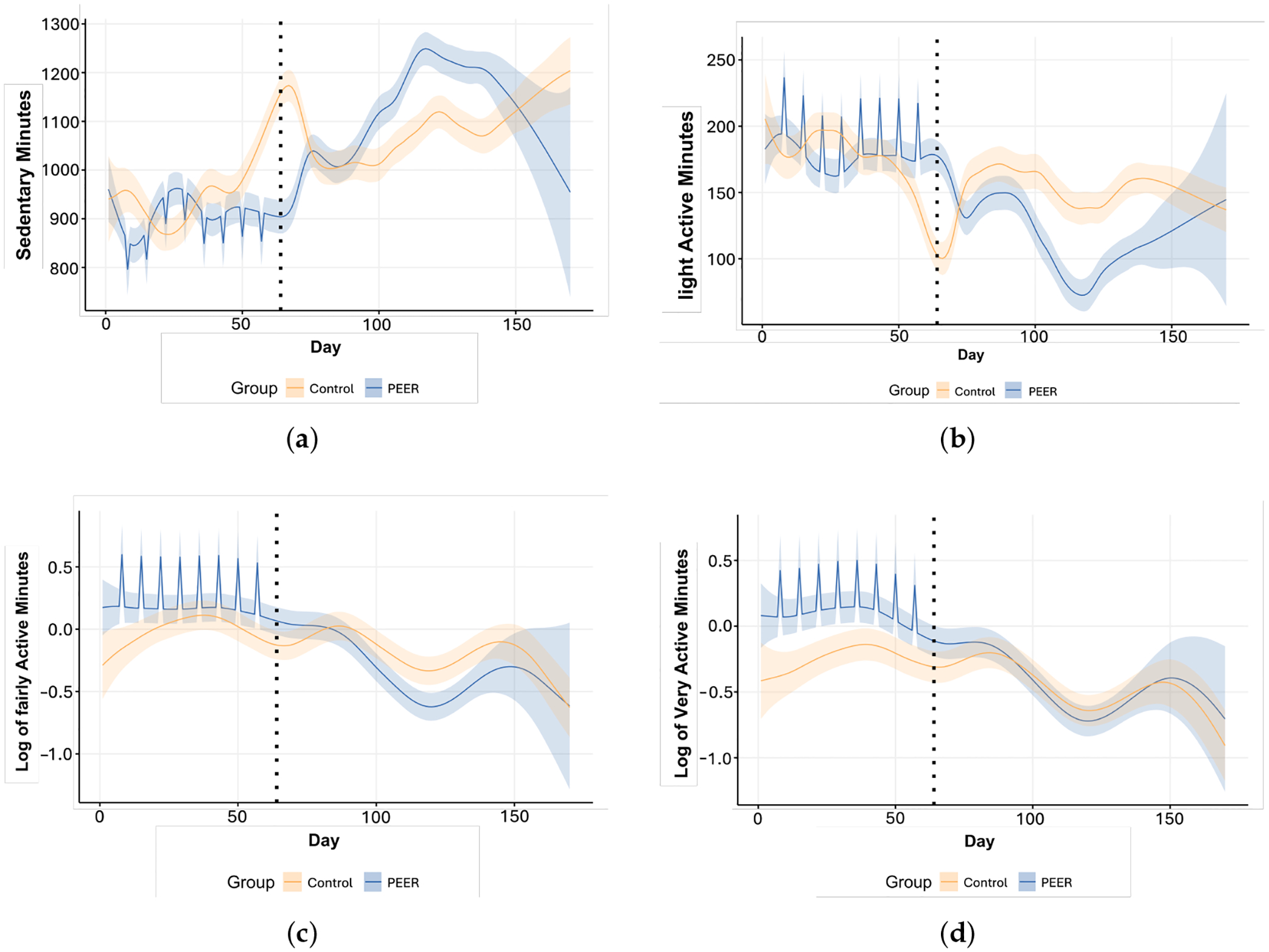
SSANOVA model prediction of daily PA by PA levels and participant groups. Blue: PEER group. Orange: control group. Light shaded area: 95% confidence interval. Black dotted line: intervention program completion and the beginning of the follow-up period. The spikes in PEER group are the group workout days in the PEER intervention program. **(a)** Sedentary minutes; **(b)** light active minutes; **(c)** fairly active minutes; **(d)** very active minutes.

To further illuminate the effectiveness of the PEER-led intervention across different PA levels, our analysis employed a partial smoothing spline model that incorporates a parametric β component for days when interventions occurred. This enhancement in our model was crucial for capturing discrete jumps in activity levels on intervention days (spikes in Figure 3), which are typically not captured by models accounting for smoother variability. A positive β value associated with a specific workout day signifies a measurable increase in PA compared to non-intervention days, directly highlighting the intervention’s impact on enhancing PA levels. Conversely, a negative β value on these days indicates a reduction in PA, suggesting that the intervention may inadvertently contribute to increased sedentary time, as observed in Figure 3a.

As shown in Figure 3a, the PEER group demonstrated a substantial reduction averaging 53.09 sedentary minutes per day (95% CI: [−130.78, 24.60]) compared to the control group during the intervention period, corresponding to a 6.7% decrease relative to their baseline sedentary time of 795 min/day, with a small effect size (Cohen’s d=-0.22), indicating a modest practical difference in sedentary time between the groups. Notably, there was no significant difference in sedentary time at baseline between the two groups (p=0.19), ensuring a fair comparison of intervention effects. This reduction was most pronounced around the midpoint of the intervention, suggesting a sustained effort to decrease sedentary behavior during this phase. The partial spline model further highlighted specific days with the most significant reductions in sedentary time, particularly those aligned with group workout sessions, underscoring the intervention’s effectiveness in promoting behavioral change during these active periods. However, the observed decrease in sedentary time gradually diminished over the 13-week follow-up period, indicating a waning effect of the intervention over time. Despite this attenuation, the PEER group consistently maintained lower sedentary time than the control group throughout the entire study period, although the magnitude of the difference decreased as time progressed.

At baseline, significant differences in fairly active and very active minutes between the PEER and control groups were observed (p=0.03 and p=0.01, respectively), necessitating a robust approach to account for these initial disparities. The SSANOVA model predictions were particularly valuable in addressing this challenge, allowing for an unbiased longitudinal analysis of intervention effects. By incorporating time-varying trends and stratifying by intervention groups, the SSANOVA framework revealed how these activity patterns evolved over the intervention period and follow-up. As shown in Figure 3, the differences in fairly and very active minutes between the PEER and control groups widened significantly during the intervention phase, particularly during group workout sessions, demonstrating the immediate effectiveness of the intervention. These findings underscore the value of time-series modeling in clinical trial data for providing a clear, dynamic view of intervention effects over time. Such an approach not only captures the immediate impact of the intervention but also provides insights into how these effects diminish or stabilize during the follow-up period, whereas static analyses lack the ability to visualize these nuanced temporal changes dynamics.

Figure 3c,d reveal significant increases in fairly active and very active minutes for the PEER group during the 8-week intervention period. Fairly active minutes peaked midway through the intervention, while very active minutes demonstrated a steadier increase throughout the program. During the intervention period, participants in the PEER groups engaged in an average of 0.15 additional very active minutes per day (95% CI: [0.08, 0.64]), reflecting a 13.8% increase from the baseline average of 1.09 minutes/day, with a small effect size (Cohen’s d=0.22). For fairly active minutes, participants engaged in an average of 0.21 additional minutes per day (95% CI: [−0.05, 0.57]), corresponding to a 2.8% increase from the baseline of 7.43 min/day, though this difference was not statistically significant (Cohen’s d=0.13).

The SSANOVA model predictions confirm that the intervention significantly boosted higher-intensity activity levels during the 8-week intervention, particularly during group workout sessions. Partial spline models further illustrate the immediate and direct impact of the intervention, with significant increases in activity levels observed on workout days. However, similar to the reduction in sedentary time, these initial increases in fairly and very active minutes diminished gradually over the 13-week follow-up period, with the PEER group converging toward the activity levels of the control group. By the end of the follow-up, the percentage increase in fairly active and very active minutes dropped to 1.2% and 4.5%, respectively, relative to their baseline levels. This trend highlights the short-term effectiveness of the intervention in promoting higher-intensity physical activity, while underscoring the need for continued support or follow-up strategies to sustain these activity levels over the long term.

In contrast, Figure 3b shows that light active minutes did not exhibit a consistently significant difference between the PEER and control groups throughout the intervention and follow-up periods. The patterns for light active minutes fluctuated over time, without a clear, sustained impact from the intervention. This variability suggests that the intervention had a less obvious effect on moderate activity levels than its more influential effects on fairly active and very active minutes.

Overall, the results demonstrate the immediate benefits of the PEER-led intervention in reducing sedentary behavior and increasing higher-intensity PA. However, the diminishing effects over the follow-up period suggest the short-term nature of the intervention, pointing to the potential need for ongoing engagement strategies to sustain the initial improvements in PA.

## 4. Discussion

This study demonstrated that a PEER-led group exercise intervention significantly increased fairly and very active minutes while reducing sedentary behavior in community-dwelling older adults during the 8-week exercise period. However, these improvements diminished over the 13-week follow-up period, indicating a decline in activity levels when the structured intervention was removed. This reduction highlights the challenges of maintaining long-term behavioral changes in PA without continued engagement. The immediate effectiveness of the intervention underscores the potential of PEER-led programs but also suggests that additional strategies are necessary to sustain these benefits over time. Overall, the intervention was highly effective in the short term, underscoring the value of structured group exercise programs in promoting PA among older adults in community settings.

Our results align with prior research indicating that exercise interventions can effectively reduce fall risk in older adults [48]. Unlike earlier studies that primarily focused on nurse-led exercise interventions [4,49], our study highlights the effectiveness of community-level PEER-led group exercise in improving physical activity (PA). In particular, the observed increases in fairly and very active minutes support existing evidence that exercise promotes PA and reduces fall risk among older adults.

Our findings highlight that interconnected physiological mechanisms, such as improved balance and muscle strength, as well as psychological mechanisms, including increased self-efficacy and cognitive reframing, drive the intervention’s success. These mechanisms work together to motivate participants to engage in PA and overcome barriers such as fear of falling. We extended the work by Xie et al. [36] and demonstrated the advantage of SSANOVA by offering a flexible framework that captures non-linear trends, quantifies temporal dynamics, and accommodates skewed distributions, which are often overlooked by the simpler traditional methods commonly used in accelerometer data analysis, such as regression and t-tests [24]. SSANOVA provides more precise, function-level inferences, and scaling efficiently to large datasets, and captures non-linear relationships, making it an ideal tool for analyzing complex biomechanical and PA data. By integrating physio-feedback, cognitive restructuring, and group-based support, the PEER intervention presents a promising model for addressing both the physical and psychological aspects of active aging, in spite of persistent challenges [50].

**Figure 4.**
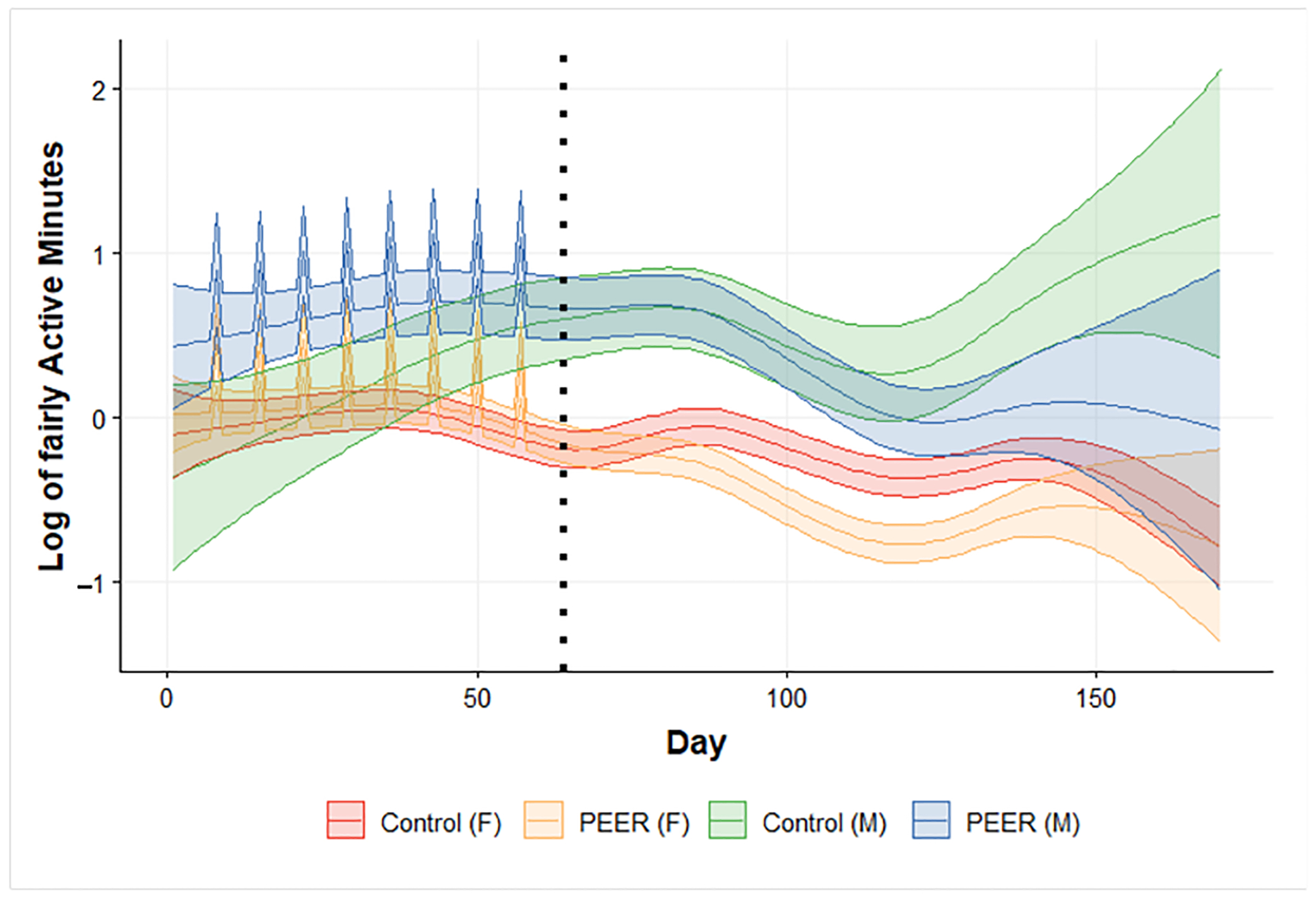
SSANOVA model prediction of log of daily very active minutes by participant group and gender. Blue: PEER male. Orange: PEER female. Green: control male. Red: control female. Light shaded area: 95% confidence interval. Black dotted line: intervention program completion and the beginning of the follow-up period. The spikes in PEER groups are group workout days in the PEER intervention program.

Despite these promising findings, we do recognize the limitations of this study. As this study presents preliminary results from ongoing research, the relatively small sample size currently affects the statistical power to detect significant differences for certain outcomes, such as fairly active minutes, where trends suggest positive effects of the PEER intervention but lack statistical significance. As the study progresses and additional participants are enrolled, we anticipate that a larger sample size will provide greater statistical power, potentially revealing more significant and robust differences between the intervention and control groups. The small sample size also limits the generalizability of the findings to the broader population of older adults. While our analysis captures overall trends, it does not yet allow for a detailed examination of subgroup-specific effects, such as potential differences in intervention outcomes across gender or racial/ethnic groups. The imbalance in the gender construct of the first two completed sites (as shown in Table 1) affects the reliability of gender-stratified modeling results, as demonstrated by our attempts to include gender in the model in Figure 4. While the figure highlights trends in gender-specific intervention effects, the 95% confidence intervals around male groups are significantly wider than those of female groups due to the lack of data from male participants. A similar situation applies to race/ethnicity, as the first two sites consist primarily of African American and Hispanic older adults. These limitations hinder our ability to draw robust conclusions about subgroup-specific effects, such as differences across gender or racial/ethnic groups. As the study progresses and additional participants are enrolled, the larger and more balanced sample size will facilitate more reliable subgroup analyses to explore these nuanced effects. Future work will leverage the complete dataset to comprehensively assess interactions between demographic factors, such as gender, and intervention outcomes, addressing this limitation.

Overall, this study highlights the effectiveness of PEER-led programs in older adults and underscores the need for approaches to foster long-term adherence to active lifestyles [51]. Beyond demonstrating short-term benefits, it is essential to consider the cost-effectiveness and scalability of such interventions to support broader implementation. Leveraging affordable, accessible technologies and integrating community-based delivery systems can make PEER interventions feasible for diverse populations, including underserved and low-income groups. Future research should address current limitations, explore mechanisms to maintain the benefits of interventions over time, and evaluate the economic viability and scalability of these programs to inform public health policies aimed at promoting sustained physical activity among older adults.

## 5. Conclusions

In this paper, we demonstrated that the PEER-led group exercise intervention was effective in increasing PA among community-dwelling older adults, specifically through increases in fairly active and very active minutes and reductions in sedentary time. SSANOVA modeling analysis revealed significant improvements during the intervention period, particularly during the group exercise sessions. However, the lack of sustained effects over the follow-up period suggests that continued support or intervention is necessary to maintain these benefits in the long term. Despite this, the intervention proved to be a valuable approach for promoting PA among older adults. Future research will investigate the intervention’s potential for fall prevention, PA pattern differences among various demographic groups, and explore strategies such as integrating mobile health applications and ecological momentary assessment (EMA) reminders into our PEER programs to extend their long-term effective impact. In clinical practice, fall prevention should emphasize a combination of technology-based fall risk assessments that include subjective and objective measures and provide feedback to older adults regarding their fall risk levels. Furthermore, healthcare providers should offer community-based fall prevention programs that can reach underserved or low-income older adults. Public health policies need to focus on enhancing physical activity, reducing sedentary behaviors, and utilizing a monitoring system along with positive reinforcement motivation strategies. These policies should involve training, educating about underlying risk factors, creating a safe home environment, and managing chronic conditions. To maximize the broader impact of such interventions, the scalability and cost-effectiveness of PEER programs must be considered. Leveraging affordable technologies and community-based approaches can make these interventions more accessible to diverse populations, including underserved groups. Future research should prioritize evaluating these aspects to inform policies aimed at sustaining long-term physical activity and promoting equitable health outcomes in older adults.

## Figures and Tables

**Table 1.** Characteristics of participants.

	Control (n = 31)	PEER (n = 33)	Overall (n = 64)
**Age (years)**			
Mean (SD)	70.8 (6.51)	75.4 (5.19)	73.1 (6.26)
Median [Min, Max]	70.4 [61.1, 85.7]	74.8 [65.9, 83.0]	73.0 [61.1, 85.7]
**Gender**			
F	29 (93.5%)	27 (81.8%)	56 (87.5%)
M	2 (6.5%)	6 (18.2%)	8 (12.5%)
**Race**			
Asian	0 (0%)	1 (3.0%)	1 (1.6%)
African American	30 (96.8%)	3 (9.1%)	33 (51.6%)
Hispanic	1 (3.2%)	20 (60.6%)	21 (32.8%)
White	0 (0%)	9 (27.3%)	9 (14.1%)
**Number of Falls**			
Mean (SD)	0.867 (1.76)	0.242 (0.502)	0.540 (1.29)
Median [Min, Max]	0 [0, 8.00]	0 [0, 2.00]	0 [0, 8.00]
Missing	1 (3.2%)	0 (0%)	1 (1.6%)
**Fall Injuries**			
0	29 (93.6%)	31 (93.9%)	50 (93.8%)
1	1 (3.2%)	2 (6.1%)	3 (4.7%)
2	1 (3.2%)	0 (0%)	1 (1.6%)

## Data Availability

We will follow NIH data share policy to share the data when the study completed.

## Abbreviations

The following abbreviations are used in this manuscript:

SSANOVA: Smoothing spline analysis of variance
PEER: Physio-fEedback Exercise pRogram
PA: Physical Activity
METs: Metabolic Equivalents
